# Challenges Associated With the Assessment of Exposure to Ageism

**DOI:** 10.1093/geroni/igaa057.2224

**Published:** 2020-12-16

**Authors:** Liat Ayalon

**Affiliations:** Bar-Ilan University, Ramay Gan, HaMerkaz, Israel

## Abstract

Ageism is defined as stereotypes, prejudice and discrimination towards people of any age. Ageism can be both positive and negative. In order for an individual to report exposure to ageism, several steps should occur: the individual has to notice the events, interpret them as ageist and report exposure to ageism. Any of these steps may go awry along the way. This presentation uses data from the European Social Survey and the Health and Retirement Study to illustrate the importance of item placement, item phrasing and respondent’s mood in responding to items concerning perceived exposure to ageism. A strong priming effect demonstrated a gap between reports of perceived exposure within the ageism module (33.7%) vs. reports within the neutral context (1.1%). A cross-lagged analyses revealed that one’s depressive symptoms are predictive of perceived exposure to ageism and not the other way around. Findings illustrate the importance of the context effect.

